# X-ray Crystallography and Electron Paramagnetic Resonance Spectroscopy Reveal Active Site Rearrangement of Cold-Adapted Inorganic Pyrophosphatase

**DOI:** 10.1038/s41598-020-61217-6

**Published:** 2020-03-09

**Authors:** Masaki Horitani, Kazuki Kusubayashi, Kyoka Oshima, Akane Yato, Hiroshi Sugimoto, Keiichi Watanabe

**Affiliations:** 1 Department of Applied Biochemistry and Food Science, Saga University, 1 Honjo-machi, Saga, Saga, 840-8502 Japan; 2 Synchrotron Radiation Life Science Instrumentation Team, RIKEN SPring-8 Center, 1-1-1 Kouto, Sayo, Hyogo, 679-5148 Japan

**Keywords:** X-ray crystallography, Metalloproteins, Enzyme mechanisms

## Abstract

Inorganic pyrophosphatase (PPase) catalyses the hydrolysis reaction of inorganic pyrophosphate to phosphates. Our previous studies showed that manganese (Mn) activated PPase from the psychrophilic bacterium *Shewanella* sp. AS-11 (Mn-Sh-PPase) has a characteristic temperature dependence of the activity with an optimum at 5 °C. Here we report the X-ray crystallography and electron paramagnetic resonance (EPR) spectroscopy structural analyses of Sh-PPase in the absence and presence of substrate analogues. We successfully determined the crystal structure of Mn-Sh-PPase without substrate and Mg-activated Sh-PPase (Mg-Sh-PPase) complexed with substrate analogue (imidodiphosphate; PNP). Crystallographic studies revealed a bridged water placed at a distance from the di-Mn centre in Mn-Sh-PPase without substrate. The water came closer to the metal centre when PNP bound. EPR analysis of Mn-Sh-PPase without substrate revealed considerably weak exchange coupling, whose magnitude was increased by binding of substrate analogues. The data indicate that the bridged molecule has weak bonds with the di-Mn centre, which suggests a ‘loose’ structure, whereas it comes closer to di-Mn centre by substrate binding, which suggests a ‘well-tuned’ structure for catalysis. Thus, we propose that Sh-PPase can rearrange the active site and that the ‘loose’ structure plays an important role in the cold adaptation mechanism.

## Introduction

Inorganic pyrophosphatase (PPase) catalyses the hydrolysis reaction of inorganic pyrophosphate (PP*i*) to two inorganic phosphates (P*i*) and plays an essential role for living organisms^1^. Soluble PPase consists of two families, I and II, which have different secondary and tertiary structures. Family I PPases are widespread organisms including bacteria and mammalians^1^, whereas family II PPases are almost exclusively found in bacteria and archaebacteria^2–4^. Both the family I and II PPases require divalent metal ions for their reactivity. All PPases are magnesium ion (Mg^2+^) dependent enzymes. Family II PPases require divalent transition metals, such as Mn^2+^ ^3,5^ or Co^2+^ ions^6^ for maximum activity.

X-ray structural analyses of family II PPases from *Bacillus subtilis* (Bs-PPase) and *Streptococcus gordonii* (Sg-PPase) have been reported^7,8^. They are homodimers and each monomer consists of two domains (N- and C-terminal domains) connected by a flexible hinge region. These two domains are in the ‘open’ state in the absence of substrate. Substrate binding results in a ‘closed’ state, with one exception^7^. Two sites (M1 and M2) for transition metal ion have been demonstrated in the active site in the ‘open’ state of Bs-PPase, while four metal ions (M1 to M4) are found in the crystal structure of Bs-PPase in complex with imidodiphosphate (PNP)^9^. Metal requirement studies for Bs-PPase have shown that the M2 site is preferred for divalent transition metal ions, such as Mn^2+^ or Co^2+^, while other metal binding sites (M3 and M4) favour Mg^2+^ ions in catalysis. However, it is still unclear whether the M1 site utilizes a transition metal or Mg^2+^ ions^4,8,10^. According to the crystal structure of Bs-PPase, the five-coordination sphere of M2 site is changed to a hexa-coordination by substrate binding. This structural change of the M2 site is considered the main reason for family II PPases requiring transition metal ions for maximum activity. In addition, a nucleophilic water coordinated with three metals (M1, 2 and 4), which is very uncommon in other hydrolysis enzymes^4,5,7,8,11^. This tri-metal coordination might cause higher activity of family II PPase than family I PPase.

We previously reported the expression and purification of family II PPase from the psychrophilic *Shewanella* sp. AS-11 (Sh-PPase) isolated from shellfish living in the Southern Ocean (Antarctic Ocean)^12^. Sh-PPase activated by Mn^2+^ ions (Mn-Sh-PPase) displayed the highest activity at 5 °C, which is characteristic of cold-adapted enzymes^12^. Our previous investigation using inductively coupled plasma-mass spectroscopy (ICP-MS) suggested the presence of two Mn^2+^ ions in the protein^12^. In addition, Sh-PPase was also activated by other transition metal ions, such as Co^2+^ and Zn^2+^, and their activities were comparable to that of Mn-Sh-PPase^13^. However, the broad metal selectivity and cold adaptation mechanism for Sh-PPase remain poorly understood due to lack of the structural information.

Electron paramagnetic resonance (EPR) spectroscopy is a powerful tool to study the structure of the mono- and di-nuclear Mn^2+^ centre of complexes and enzymes in solution. Several enzymes containing a di-Mn^2+^ active site have been reported. These include arginase^14,15^, catalase^16,17^, prolidase^18^ and thiosulfate-oxidase^19^. In addition, some di-Mg^2+^ and di-Zn^2+^ enzymes in native form retain their activity when substituted with di-Mn^2+^ ions, including *S*-adenosylmethionine synthetase^20^, concanavalin A^21^, enolase^22^, ribonucleotide reductase^23,24^ and ribonuclease H^25^. In almost all these enzymes for which crystal structures have been determined, the divalent metals were bridged by a water/hydroxide and one or two carboxylates from aspartate/glutamate residues and the remaining ligands of each metal were coordinated with oxygen residues of carboxylate and/or nitrogen residues of histidine.

**Figure 1:**
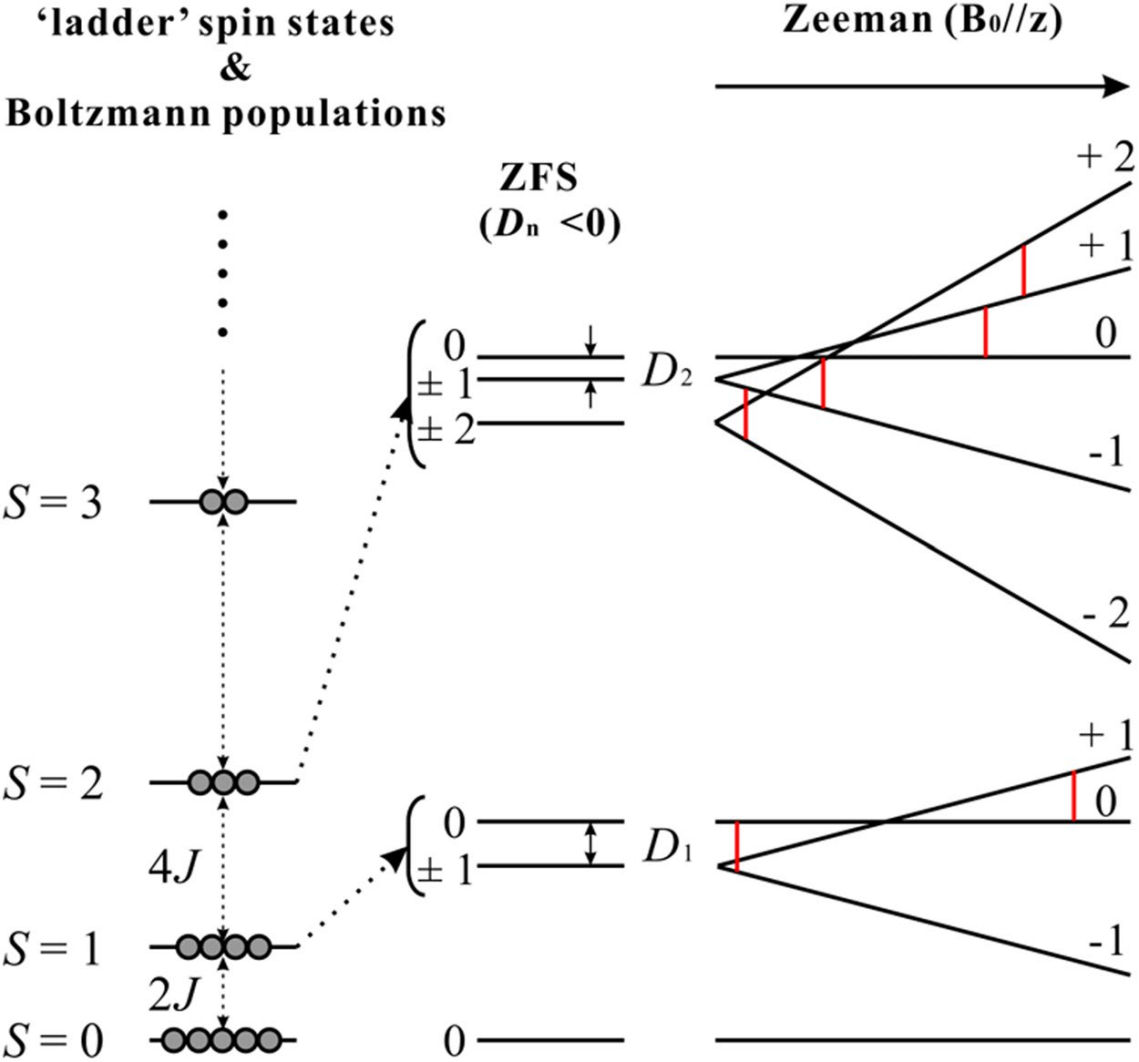
The ‘ladder’ electronic structure of the di-Mn^2+^ system, Boltzmann populations and Zeeman splitting with ZFS along the z-axis. The circles represent the number of populations. The red lines denote possible EPR transitions. The sign of axial ZFS parameters were assumed to be negative.

Such di-Mn^2+^ centres (two ^55^Mn, 3*d*^5^ high-spin; *S*_1_ = *S*_2_ = 5/2, *I*_1_ = *I*_2_ = 5/2) experience a weak antiferromagnetic exchange coupling, which yields total spin *S* = 0 in the ground state and *S* = 1, 2, … 5 in the excited states (Fig. 1) as described by Howard *et al*.^26^ and Golombek *et al*.^27^. These are referred to as ‘ladder’ spin states (Fig. 1, left). The singlet ground state is EPR-silent, whereas all excited states have several allowed EPR transitions due to much smaller zero field splitting (ZFS) than the microwave quantum energy (Fig. 1, right) with 11 characteristic hyperfine splitting in the EPR spectrum by two equivalent *I* = 5/2 nuclear spins. The overall EPR spectra of these enzymes and model compounds are complicated, which makes analysis of the spectra difficult. Moreover, the relationship between EPR-derived parameters and structure is also unclear. Dismukes *et al*. developed a procedure for the direct estimation of the di-Mn distance based on an empirically linear correlation between the Mn-Mn distance and the axial ZFS parameter *D*_2_ from the quintet state (*S* = 2)^28^. Using this correlation, the deviation of the EPR determined Mn-Mn distance was within ±0.03 Å compared with known X-ray determined model structures^28–36^.

In this paper, we initially examined the signal from first excited state (*S* = 1). The exchange coupling constant (*J*) was then determined from the temperature dependence of EPR signals from *S* = 1. Finally, the signal from *S* = 2 was derived from the Boltzmann curve of *S* = 2 and the Mn-Mn distance of Sh-PPase in solution was determined. X-ray crystallography and EPR spectroscopy were used for the analysis of the overall and active site structural change of Sh-PPase induced by the substrate. The findings reveal the cold adaptation mechanism in Sh-PPase.

## Results and Discussion

### X-ray crystal structure

**Figure 2:**
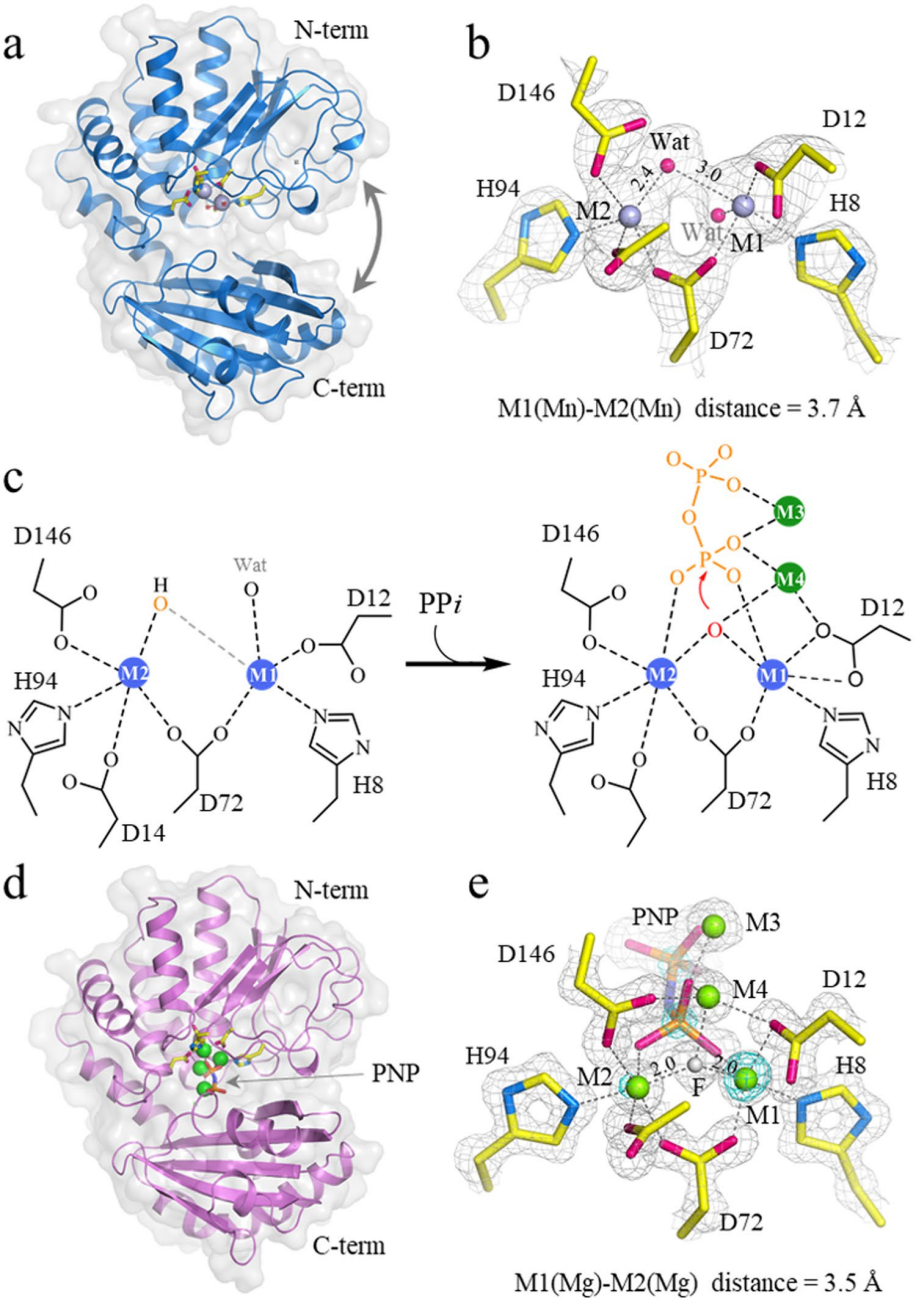
Crystal structure of Sh-PPase in the absence and presence of the substrate analogue. (**a**) Overall structure of Mn-Sh-PPase. (**b**) Coordination sphere of M1 and M2 sites of Mn-PPase. (**c**) A diagram of the di-Mn centre in Sh-PPase. The metal sites are shown in circles. The bridged water is bound with three metals in the presence of substrate (M1, M2 and M4). (**d**) Overall structure of Mg-Sh-PPase complexed with PNP. (**e**) Coordination sphere of M1, M2, M3 and M4 of Mg-Sh-PPase with PNP. The bridged water/fluoride and metals are shown as spheres and PNP and amino acid residues as sticks. The 2Fo-Fc omit map is also shown as grey mesh (1.4 σ and 2.0 σ for Mn-Sh-PPase and Mg-Sh-PPase, respectively). The cyan-coloured mesh denotes the anomalous difference Fourier map (5 σ). Metals are shown as spheres and PNP and amino acid residues of coordination sphere of active site are shown as stick models.

The crystal structure of Mn-Sh-PPase in the absence of substrate was determined at 2.2 Å resolution (Fig. 2a). The M1 and M2 sites showed similar coordination spheres to other family II PPases (Fig. 2b,c). The M1 site in Mn-Sh-PPase was coordinated with one nitrogen of histidine, two oxygens of aspartates (D12 and D72), and a bridged water. Additionally, the M1 site was bound with an extra water molecule^8^. Coordination geometry of the M2 site in Mn-Sh-PPase was also similar with that of Bs-PPase and Sg-PPase with 5-coordinated bipyramidal geometry. However, the distances of a bridged water in the Sh-PPase from M1 and M2 (3.0 and 2.4 Å, respectively) were considerably longer compared with crystal structures of other di-Mn enzymes (Table 1). In addition to the Mn-O distance, the Mn-Mn distance of Sh-PPase was also longer than arginase and catalase.

**Table 1:** Active site and electronic structure of di-Mn enzymes.

	Metal-O (bridged) distances (Å)^a^	*J* (cm^−1^)	*D*_2_ (cm^−1^)	Metal-Metal distance (Å)		Reference
				EPR	X-ray^a^	
Sh-PPase	3.0, 2.4	−0.85	−0.0575	3.57	3.7	this work
Sh-PPase (+PNP)	2.0, 2.0	−1.3	−0.07	3.52	3.5	this work
Sh-PPase (+sulphate)	—	−1.2	−0.07	3.52	—	this work
Bs-PPase	2.4, 1.8^b^	—	—	—	3.7^b^	^7^
Bs-PPase (+PNP)	2.0, 2.0^c^	—	—	—	3.5^c^	^9^
Sg-PPase	2.1, 2.1^d^	—	—	—	3.7^d^	^7^
Arginase	2.4, 2.4^e^	—	−0.056	3.57	3.4^e^	^15^
Arginase (+substrate)	2.3, 2.0 ^f^	−2.0	−0.073	3.50	3.3 ^f^	^26^
Catalase (+phosphate)	—	−5.6	−0.051	3.59	—	^26^
Concanavalin A	2.2, 2.6^g,h^	−1.8	—	—	4.3^g^	^21^

^a^Average of subunits.

^b,c,d,e,f,g^Data were taken from PDB ID; 1K23, 2HAW, 1K20, 1RLA, 1HQG, 1DQ6, respectively.

^h^Distance between Mn and carboxylate oxygen of D10.

A previous crystallographic study of Bs-PPase showed that the addition of the fluoride ion in the PPase substitutes for the bridged water and dramatically reduces the reaction rate^9^. Therefore, we attempted to crystallise the PNP-bound form of Sh-PPase in the presence of sodium fluoride. However, the crystals obtained did not show any electron density for PNP, likely because Mn-Sh-PPase only weakly degrades PNP, even in the presence of sodium fluoride. In contrast, Sh-PPase activated by Mg^2+^ ions (Mg-Sh-PPase) has much lower catalytic activity with PP*i* or PNP than Mn-Sh-PPase^13^, and we obtained well-diffracted crystals of Mg-Sh-PPase in the presence of PNP and sodium fluoride. X-ray diffraction data at 1.3 Å resolution were obtained. The electron density unambiguously showed that the PNP and four Mg^2+^ atoms were bound to the di-metal centre of Sh-PPase. The overall structures of Sh-PPase with and without substrate were very similar with other family II PPases, and that the binding of substrate analogue induced the conformational change from the ‘open’ to ‘closed’ state (Fig. 2a,d), as observed in Bs-PPase^7^. In the crystal structure of Mg-Sh-PPase with PNP, the bridged water between Mg^2+^ ions at M1 and M2 is assumed to be replaced by a fluoride ion (Fig. 2e). An anomalous difference Fourier map indicated that M1 and M2 metal sites in the crystal structure of Mg-Sh-PPase contain a small fraction of metals other than Mg^2+^. As shown below, the EPR spectrum for apo Sh-PPase at 15 K showed an unexpected signal at *g*~4.3, which is characteristic of mono Fe^3+^ ion^37^ (see EPR results). This signal was considerably reduced by the addition of Mn to Sh-PPase, indicating that contaminated Fe^3+^ was replaced by Mn^2+^. These observations suggest that Fe^3+^ ion is contaminated in the active site of apo and Mg-Sh-PPase, even though all buffers were treated using ethylenediamine- *N,N,N*′*,N*′-tetraacetic acid (EDTA). The peak height of the anomalous difference map for M1 and M2 showed similar density level with those of sulphur atoms of Cys or Met residues. Based on the anomalous scattering factor of these elements (*f* ″ = 0.24 for S; *f* ″ = 1.6 for Fe) at an X-ray wavelength of 1.0 Å, M1 and M2 were occupied by Fe with an estimated content of 15% and 7%, respectively. Similarly, it was reported that M1 and M2 sites of the crystal structure of Bs-PPase with PNP and fluoride ion were also occupied by Fe/Mn in a 6/1 ratio^9^.

As mentioned above, a unique structural feature of Mn-Sh-PPase found in the present X-ray analysis is the longer distance between bridged water and two Mn atoms compared with that in other family II PPases (Table 1). In the crystal structure of Mg-Sh-PPase complexed with PNP, the site of bridged water was replaced by an oxygen atom of PNP. The active site of Mg-Sh-PPase complexed with PNP shows common coordination sphere, like other family II PPases^4,9^. The M1 site was converted to the 6-coordinated slight rhombic octahedral geometry by binding of PNP, and the M2 site was changed to a nearly axial octahedral coordination sphere (Fig. 2c,e). Based on the crystal structure of Bs-PPase complexed with PNP^9^, Fabrichniy and co-workers proposed that such a dynamic coordination change by binding of substrate must be why family II PPases prefer transition metals to Mg^2+^ ions^8^. In addition, a bridged water in the crystal structure of Mg-Sh-PPase with PNP, as well as Bs-PPase, shows the formation of unique tri-metal coordination sphere (Fig. 2c,e), where it is bound with 3 metal ions (M1, M2 and M4). This unique tri-metal coordination should occur with all 3 lone pairs on the oxygen atom of a nucleophilic hydroxide anion (Fig. 2c). The tri-metal coordination needs to play an important role to hold a nucleophile at the suitable position and activate a bridged water for nucleophilic attack. We propose that the tri-metal coordination would be broken, most probably at a bond with M4, because of its longer distance, so that the oxygen atom of nucleophilic hydrogen oxide becomes able to attack the phosphorus of inorganic pyrophosphate.

### CW X-band EPR measurements

**Figure 3:**
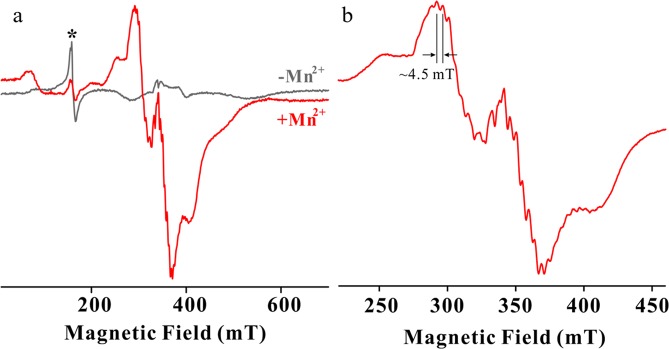
CW X-band EPR spectra for Sh-PPase before/after Mn^2+^ activation. (**a**) Mn-Sh-PPase was prepared by the incubation into the buffer in the presence of excess of Mn^2+^ ions at 4 °C for 2 hours, and excess Mn^2+^ ions were removed by buffer exchange and subsequent application to a desalting column (red line). EPR spectrum of apo Sh-PPase is shown as a grey line. The asterisk signal was free ferric ions at *g*~4.3. (**b**) EPR spectrum of Mn-Sh-PPase near *g*~2 showed several sets of 11 hyperfine splittings (~4.5 mT) characteristic of the di-Mn centre. The conditions used were: microwave frequency = 9.597 GHz, microwave power = 2 mW, 100 kHz modulation amplitude = 10 G and temperature = 15 K.

Purified Sh-PPase has no transition metal in the active site (apo Sh-PPase). The apo Sh-PPase was incubated at 4 °C for 2 hours with the buffer containing 100 mM Tris-HCl (pH 7.0) and 15 mM MnCl_2_. Subsequently the excess Mn^2+^ ions were removed by dialysis and buffer exchange using an ultrafiltration device. The continuous-wave (CW) X-band EPR spectrum showed a strong signal at *g*~2 with 6 hyperfine splitting (~9 mT) of a characteristic mono-nuclear Mn^2+^ centre^38,39^ (see Supplementary Fig. S1). The signals split by ~4.5 mT at ~290 mT (see Supplementary Fig. S1 inset) was also observed, which was characteristic of di-Mn^2+^ centre (two equivalent *I* = 5/2)^18,19,26–28^. The signal of mono-nuclear Mn^2+^ was disappeared by additional buffer exchange of the sample by metal-free buffer using desalting spin column. Figure 3a shows CW X-band EPR spectra of apo- and Mn-Sh-PPase at 15 K. Addition of Mn ion to apo Sh-PPase diminished mono Fe^3+^ signal at *g*~4.3 (Fig. 3a, asterisk). Alternatively, several hyperfine splitting with ~4.5 mT appeared in a wide range of the spectrum (Fig. 3b). This result confirmed that the active site of Sh-PPase has dominantly di-Mn centre and is consistent with our earlier ICP-mass results in which two metal binding sites of M1 and M2 are occupied by transition metals^12^.

### Determination of antiferromagnetic coupling

**Figure 4:**
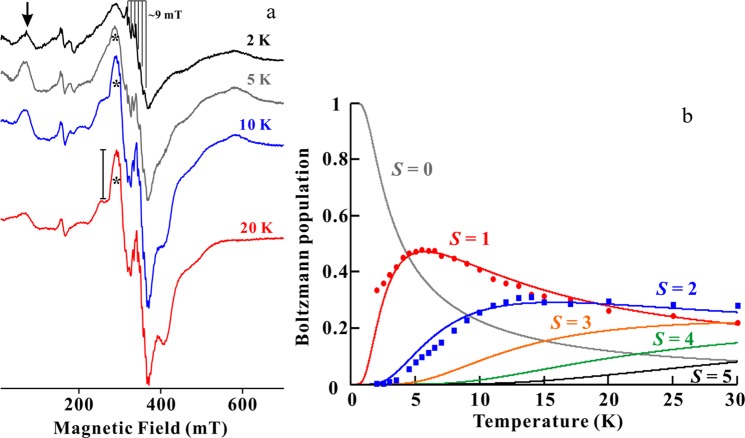
Temperature dependence of CW X-band EPR spectra and the Boltzmann curves for Mn-Sh-PPase. (**a**) The temperatures were as indicated in Fig. 4a. The arrow and asterisk indicate EPR transitions from *S* = 1 and *S* = 2, respectively. Conditions were the same as Fig. 3. (**b**) The temperature dependence of the EPR signals from *S* = 1 (red circles) and *S* = 2 (blue squares) and calculated Boltzmann population (lines). The best fit antiferromagnetic coupling constant, *J*, is −0.85 ± 0.05 cm^−1^. The Boltzmann curve is drawn with *J* = −0.85 cm^−1^. The experimental intensities are shown in red circles and blue squares.

Figure 4a shows the temperature dependence of the EPR spectra for Mn-Sh-PPase. Since EPR spectra at *g*~2 were overlaid with signals contributed from several exited states of the ‘ladder’ di-Mn spin system, it was difficult to deconvolute the individual signals. EPR spectra at 2 K still showed a strong signal at ~350 mT with 6 hyperfine splitting of ~9 mT. The intensities of EPR signals for di-Mn centre at 2 K should be much smaller because the Boltzmann population was mostly in the ground state (*S* = 0), which is EPR-silent. This suggested that some of the mono-nuclear Mn^2+^ ions remained in the sample, whose intensity are markedly increased at a low temperature. We propose that Mn^2+^ ions that cannot be removed by non-specific binding to protein or mono-nuclear centre in the active site of Sh-PPase exist in Mn-Sh-PPase as a minor component.

We first analysed a well-isolated peak at the lowest field as indicated by arrow in Fig. 4a. The red circles in Fig. 4b are plots of the double integrated intensity of these signals with temperature. To obtain the accurate exchanged coupling constant (*J*), the absolute intensity was normalised appropriately. These signals reached the maximum intensity at ~6 K. If these signals would be the transition from *S* = 2 or higher spin system, the signals from *S* = 1 whose intensity reach the maximum at below 6 K must be appeared (Figs. 1 and 4b). No such signal, however, was observed below 6 K, except for the signals from mono-nuclear Mn^2+^ as an impurity. These observations led us to suggest that this signal (marked by the arrow) was originated from *S* = 1 state. The Boltzmann curve for *S* = 1 was calculated by Eq. 3 and the best fitted curve was given an exchanged coupling constant, *J* = −0.85 ± 0.05 cm^−1^ (Fig. 4b. red line). This value was the smallest compared with other di-Mn enzymes (Table 1)^21,28^. Most importantly, this is consistent with the X-ray results. In the crystal structure of Mn-Sh-PPase in the absence of substrate, the distances between bridged water and M1/M2 were considerably longer than other di-Mn enzymes, including other family II PPases. This longer metal-O (bridged) distances yield a considerably weak antiferromagnetic coupling in the di-Mn centre. Our observations reveal that the di-Mn centre of Mn-Sh-PPase in both single crystal and solution forms has longer distance with bridged water. This suggests that the active site of Mn-Sh-PPase has an intrinsically ‘loose’ structure. Cold-adapted enzymes, in general, is thought to have flexible structure so that it can maintain the perturbation for the reactivity even at cold temperature. We conclude that the ‘loose’ structure of the active site in Mn-Sh-PPase allows binding of substrate and conformational change of active site required for the catalysis at low temperature, which is a key to cold adaptation mechanism of Sh-PPase.

### Determination of axial ZFS parameters for S = 2 and di-Mn distance

EPR-derived parameters were rarely directly combined with the structural information. Dismukes *et al*. developed a procedure to directly estimate the Mn-Mn distance from an empirically linear correlation between Mn-Mn distances and the axial ZFS parameters *D* (*D*_2_) from *S* = 2^28^. As shown in Figs. 3 and 4a, however, it was difficult to assign signals from *S* = 2 due to the overlaying of several EPR transitions. To identify the signals from *S* = 2, the intensities of temperature dependence were plotted and compared with *S* = 2 Boltzmann curve with *J* = −0.85 cm^−1^ (Fig. 4b, blue line). The temperature dependence of the peak marked with the asterisk in Fig. 4a was the best fit with the curve of *S* = 2 (Fig. 4b, blue squares). These signals were overlaid with some signals from other transitions of the di-Mn centre and mono-nuclear Mn impurity, so the intensities plotted in Fig. 4b were simply obtained from the peak height.

**Figure 5:**
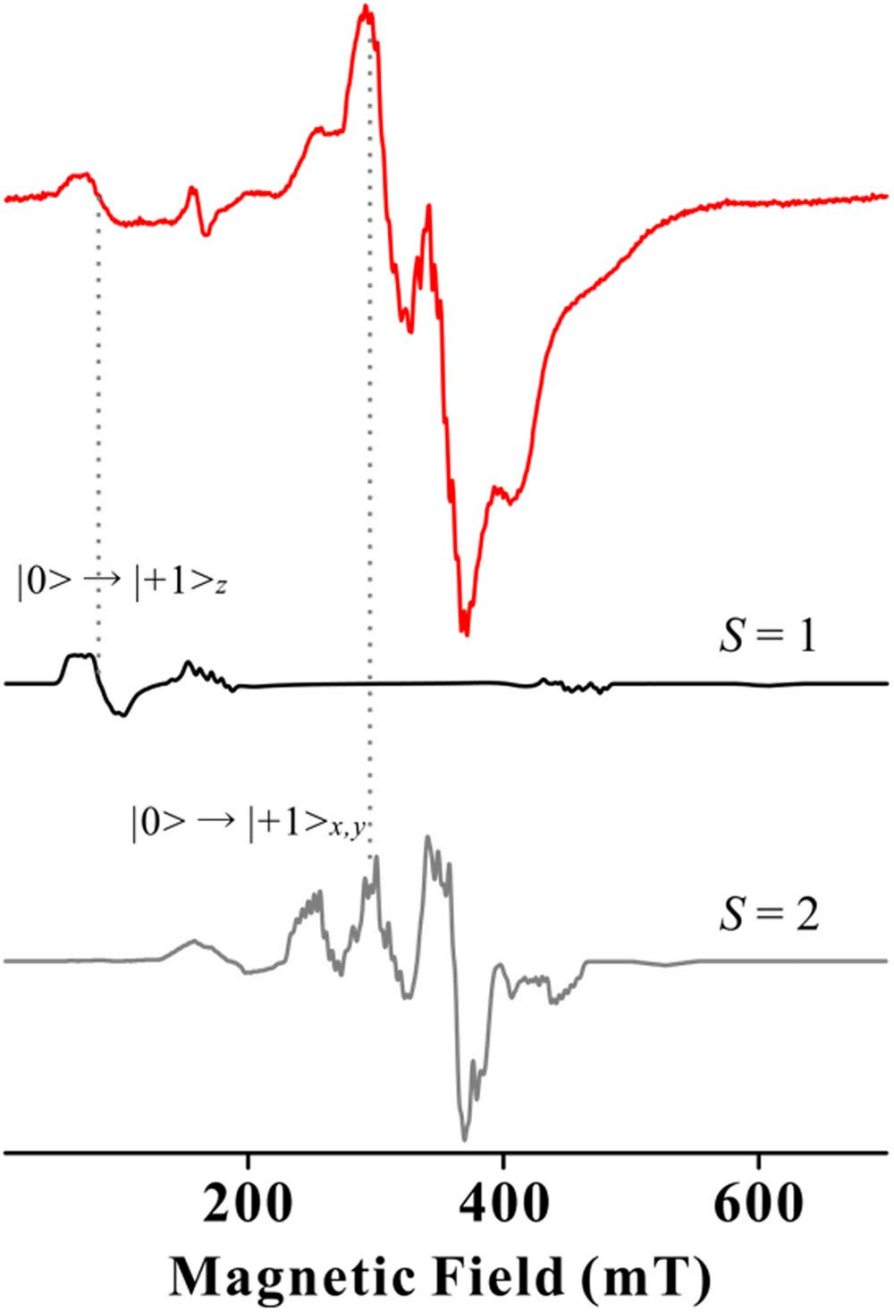
X-band CW EPR spectra at 15 K for Mn-Sh-PPase and simulation for *S* = 1 and 2. Experimental spectrum and simulation for *S* = 1 and *S* = 2 are shown as red, black and grey lines, respectively. The peak positions of |0 > ⇔|+1 > _*z*_ transition from *S* = 1 and |0 > ⇔|+1 > _*x,y*_ transition from *S* = 2 are indicated in grey dotted lines. The best fit simulation was calculated by the axial ZFS parameter, *D*_1_ = 0.248 ± 0.010 cm^−1^ and *D*_2_ = −0.0575 ± 0.002 cm^−1^_._ Experimental conditions are the same as in Fig. 3.

We simulated EPR spectra using spin Hamiltonian treated with single spin system, such as *S* = 1 and 2, as calculated using Eq. 2. The overall pattern of the EPR spectra could not reproduced by simulation from only *S* = 1 and 2 because experimental spectra were contributed from higher exited states of the di-Mn centre (*S* = 3, 4 and 5) and mono-Mn impurity. Since the estimation for the magnitude of *D*-value is sensitive to the peak position, we used EPR simulation to fit the peak position with experimentally assigned peaks of *S* = 1 and 2. The best fit simulation for *S* = 1 and *S* = 2 as shown in Fig. 5 and Supplementary Fig. S2 were given the axial ZFS parameters, |*D*_1_ | = 0.248 ± 0.010 cm^−1^ and *D*_2_ = −0.0575 ± 0.002 cm^−1^, respectively. According to the linear correlation between Mn-Mn distances of X-ray crystal structures and the axial ZFS parameters for *S* = 2, the *D*_2_ value was given to 3.57 ± 0.03 Å for the Mn-Mn distance of the Sh-PPase, which is comparable to most of other di-Mn enzymes and is within the range of linear correlation (Table 1, and see Supplementary Fig. S3)^21,28^. The EPR-derived Mn-Mn distance of Mn-Sh-PPase was comparable with the X-ray-derived distance of all family II PPases. In addition, we prepared two more samples of Mn-Sh-PPase and collected EPR spectra at 15 K (see Supplementary Fig. S4). The experimental errors were estimated for peak positions and *D*_2_ for ~0.9 mT and 0.001 cm^−1^, respectively. This error is corresponded to ~0.004 Å Mn-Mn distance, which is within a deviation of the empirically linear correlation between *D*_2_ and Mn-Mn distance (±0.03 Å).

### Substrate binding effect for Sh-PPase

**Figure 6:**
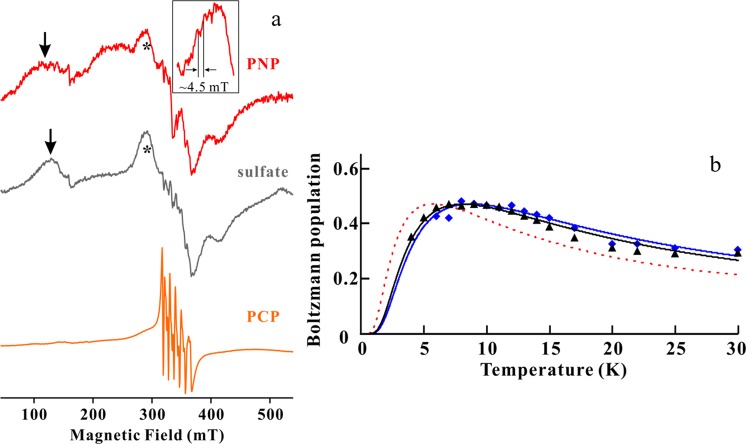
X-band CW EPR spectra at 15 K and Boltzmann populations for Mn-Sh-PPase with substrate analogues. (**a**) EPR spectra of Mn-Sh-PPase with PNP, (NH_4_)_2_SO_4_ and PCP are shown in red, grey and orange lines, respectively. The peak positions of *S* = 1 and *S* = 2 are indicated by arrows and asterisk, respectively. Inset shows an expanded signal from *S* = 2 of Mn-Sh-PPase complexed with PNP at 40 K. Experimental conditions were the same as Fig. 3. (**b**) Boltzmann population for Mn-Sh-PPase with PNP and ammonium sulphate. The temperature dependence of the EPR signals from *S* = 1 of PNP-bound and sulphate-bound are shown as blue diamonds and black triangles, respectively. The best fit calculated Boltzmann population curve of PNP-bound and sulphate-bound is shown in blue and black lines, respectively. The best fit antiferromagnetic coupling constant *J* is −1.3 ± 0.1 cm^−1^ for PNP-bound and −1.2 ± 0.1 cm^−1^ for sulphate bound. The Boltzmann curve of substrate unbound with *J* = −0.85 cm^−1^ was also drawn in dash line as a comparison.

Family II PPase only weakly converts the substrate analogue PNP to P*i*^4,9^. Therefore, for the EPR measurement, enzyme concentration was decreased to ~100 µM to prevent consumption of PNP. All samples of Mn-Sh-PPase with substrate analogues were prepared by taking the sample from the same aliquot as that for Mn-Sh-PPase in the absence of substrate analogue and their concentration was decreased by addition of the buffer without any metals. Mn-Sh-PPase were then mixed with the solution with saturated PNP or methylendiphosphonic acid (PCP) in an EPR quartz tube and transferred to liquid nitrogen within 30 seconds. The CW X-band EPR spectrum at 15 K for Mn-Sh-PPase in the presence of PNP is denoted in red in Fig. 6a (red). The overall line-shape was slightly changed. In particular, the signal from *S* = 1 (Fig. 6a, arrow) was broadened and shifted to a higher magnetic field compared with Mn-Sh-PPase without substrate, indicating that the *D*_1_ value became smaller. Importantly, the characteristic 45 mT hyperfine structure of the EPR spectrum at 40 K for the Mn-Sh-PPase with PNP was evident, even at a lower concentration of Mn-Sh-PPase (Fig. 6a, inset). This confirmed that the solution of Sh-PPase had a di-Mn centre in the active site in the presence of substrate, while the crystal structure was unresolved for Mn-Sh-PPase with substrate. The antiferromagnetic coupling constant of Mn-Sh-PPase with PNP was estimated by the signal intensity of *S* = 1, yielding *J* = −1.3 cm^−1^, which was larger than the value for Mn-Sh-PPase without substrate (Fig. 6b, Supplementary S5a and Table 1). In addition, the signal from *S* = 2 of Mn-Sh-PPase complexed with PNP was shifted to ~9 mT lower magnetic field than that of substrate unbound enzyme (see Supplementary Fig. S6). This shift yielded a larger *D*_2_ value, which corresponded to a slightly shorter Mn-Mn distance (3.52 Å) according to an empirically linear correlation (see Supplementary Fig. S3). Since the Mn-Mn distance changed only marginally, the larger magnitude of the *J* value for Mn-Sh-PPase complexed with PNP suggested that a bridged water was closer to the di-Mn centre when substrate bound. In fact, the crystal structure of Mg-Sh-PPase with PNP displayed a shorter distance between M1/M2 and the bridged fluoride ion. This active site rearrangement is required for efficient reactivity. Our observations support the conclusion that the ‘loose’ structure of the active site is induced to a ‘well-tuned’ structure that is necessary for reactivity by binding of substrate. Hence, cold-adapted Sh-PPase is able to bind the substrate at a cold temperature due to the ‘loose’ active site. Once the substrate is bound, rearrangement of active site occurs simultaneously with the ‘well-tuned’ structure for nucleophilic attack.

The Southern Ocean is considered the largest high-nutrient low-chlorophyll (HNLC) region, with lower concentrations of minerals, such as transition metals. The ‘loose’ structure of Sh-PPase might be concerned with facilitating broad metal selectivity for Sh-PPase. This might confer a survival advantage on *Shewanella* sp. AS-11 in the metal-limited Southern Ocean.

In the sulphate-bound crystal structure of family II PPase from *Streptococcus gordonii* (Sg-PPase), sulphate ion bound in the substrate binding site and its conformation was in the ‘closed’ state like the substrate-bound form, indicating that sulphate ions might mimic the substrate or product in the active site^7^. To validate that sulphate ion can also bind to the substrate binding site of Sh-PPase, ammonium sulphate was mixed with Mn-Sh-PPase and EPR measurements were performed. Since ammonium sulphate was not the real substrate, higher concentration of enzyme could be used for EPR measurements. The EPR spectrum for Mn-Sh-PPase with sulphate ion was almost identical to that for PPase with PNP (Fig. 6a, grey). 45 mT hyperfine splitting at wide range of the spectrum was also observed, suggesting that the di-Mn centre was maintained. The antiferromagnetic coupling constant of Mn-Sh-PPase with sulphate was also estimated by the signal intensity of *S* = 1, yielding *J* = −1.2 cm^−1^ (Figs. 6b, S5b and Table 1) and the signal position of *S* = 2 (see Supplementary Fig. S6) was also identical with bound PNP. These observations suggested that sulphate ion bound correctly to the substrate binding site. We also performed EPR measurement for Mn-Sh-PPase complexed with another substrate analogue, PCP, which is used as the inhibitor for family II PPases^40^. The EPR spectrum for Mn-Sh-PPase with PCP clearly showed strong 6 hyperfine splitting characteristic of the mono-Mn^2+^ ion, and no signal was observed from the di-Mn centre (Fig. 6a, orange). These observations suggested that binding of PCP induced the decomposition of the di-Mn centre. Thus, the inhibitory mechanism of PCP was different from that of PNP. In addition, the line-width of the EPR spectra of Mn-Sh-PPase with PNP and sulphate ion were somewhat broadened, suggesting that binding of substrate increased the perturbation of the di-Mn centre. Thus, we propose that the increased perturbation of the metal sites by substrate binding plays an important role for activation of bridged water for nucleophilic attack to the PP*i* substrate.

## Conclusions

X-ray crystallography and EPR spectroscopy were used to investigate a cold-adapted family II Sh-PPase from a psychrophilic bacterium that lives in the Southern Ocean. We successfully determined the crystal structure of Mn-Sh-PPase and Mg-Sh-PPase complexed with PNP. The overall structure consisted of N- and C-terminal domains, and the overall conformation was changed from the ‘open’ to ‘closed’ state by the binding of substrate, as occurs with other family II PPases. EPR analysis revealed that the active site in Sh-PPase with and without substrate had a di-Mn^2+^ centre. The Mn-Mn distance was estimated to be 3.57 and 3.52 Å for Mn-Sh-PPase alone and complexed with substrate analogues, respectively. The most important observation was that the di-Mn centre of Mn-Sh-PPase in the absence of substrate experienced a considerably weak exchange coupling, which was increased by the binding of substrate analogues. In the X-ray crystal structure of Mn-Sh-PPase, the bridged water was further away from the di-Mn centre, and it was placed near the di-Mn centre in Mg-Sh-PPase with PNP. These observations suggest that the active site of Sh-PPase is a ‘loose’ structure and that the rearrangement of the active site upon binding of substrate generates a ‘well-tuned’ structure. Thus, we conclude that the ‘loose’ active site in Sh-PPase plays an important role for binding of the substrate at low temperature. This structural character is an advantage for the adaptation of Sh-PPase to a cold environment, such as the Southern Ocean, since the enzyme must maintain flexibility for catalysis at a cold temperature. Once the substrate is bound, the active site is changed to the ‘well-tuned’ structure that is necessary for the activation of nucleophilic water. Additionally, we propose that the ‘loose’ structure also facilitates the use of a wide range of metals, which confers a survival advantage on *Shewanella* sp. AS-11 to survive in the metal-limited condition of the NHLC region. In summary, X-ray crystallography and EPR spectroscopy allows viewing of the active site structure in Sh-PPase in both the single crystal and solution forms, and the rearrangement of the active site upon substrate binding. The present findings reveal that the ‘loose’ structure of Sh-PPase is important in the cold adaptation mechanism.

## Methods

### Preparations of Mn-activated Sh-PPase

Recombinant Sh-PPase was expressed in *Escherichia coli* BL21 (DE3) as described previously^12^. Metal-free Sh-PPase was purified from cell extracts by ammonium sulphate fractionation (2 mM EDTA and 30 mM MOPS buffer, pH 7.0) and anion-exchange chromatography (Hi-Trap Q HP column, GE Healthcare Life Science) using 20 µM EDTA, 500 mM KCl and 30 mM MOPS buffer (pH 7.0).

For EPR samples, Mn-Sh-PPase was obtained by diluting 250 µL of a solution of Sh-PPase in metal-free buffer (30 mM MOPS [pH 7.0], 2 mM EDTA) in 12 mL of 100 mM Tris-HCl buffer (pH 7.5) containing 15 mM MnCl_2_, 15 mM MgCl_2_, 50 mM KCl and 20 µM EDTA and incubating the mixture for 2 hours at 4 °C. Subsequently, excess Mn^2+^ ions were removed by dialysis with 1,000 times the volume of buffer and centrifugal ultrafiltration devices with transition metal-free buffer (100 mM Tris [pH 7.5], 15 mM MgCl_2_, 50 mM KCl and 20 µM EDTA). However, free Mn^2+^ ions still remained in the solution as indicated by a strong EPR signal with 6 hyperfine structure at *g*~2, which was characteristic of the mono-Mn^2+^ ion (see Supplementary Fig. S1). To completely remove free or non-specifically bound Mn^2+^ ions, the Mn-Sh-PPase protein was applied to the desalting spin column (APRO Science) that was equilibrated using a buffer comprised of 100 mM Tris-HCl (pH 7.5), 15 mM MgCl_2_, 50 mM KCl and 20 µM EDTA. After this treatment, the EPR spectrum was predominated by a complicated 11 hyperfine splitting characteristic of the di-Mn centre. The concentration of the enzyme was calculated from the absorbance at 280 nm (ε_280 nm_ = 23.3 mM/cm). PNP and PCP (both from Sigma-Aldrich) were used as an analogue of the substrate. As a note, Bs-PPase is also able to convert PNP, though its rate is much slower (~10^4^–10^5^ times) than that for PP*i*^4,9^. Mn-Sh-PPase for substrate binding studies were used from the same aliquot as that for the unbound substrate, but the final protein concentrations were reduced to prevent the consumption of substrate analogues. Mn-Sh-PPase was transferred into a quartz EPR tube, mixed with analogues in the tube, and then frozen in liquid nitrogen within 30 seconds.

### Crystallisation

Fractions of Sh-PPase eluted from anion-exchange chromatography were applied to a Superdex 75 gel-filtration column (GE Healthcare Life Science) equilibrated with 20 µM EDTA, 30 mM MgCl_2_, 200 mM KCl and 100 mM Tris-HCl (pH 7.5) buffer. Activation of the enzyme by Mn^2+^ was performed using the same procedure detailed above. For the Mg^2+^- and PNP-bound forms, gel-filtration fractions of metal-free Sh-PPase were collected and incubated in 20 µM EDTA, 30 mM MgCl_2_, 200 mM KCl and 100 mM Tris-HCl (pH 7.5) buffer for 2 hours at 4 °C. The protein solution was mixed with PNP and sodium fluoride at final concentrations of 5 mM and concentrated by ultrafiltration (Merck-Millipore) using a 10 kDa cut-off.

Crystals were obtained by the sitting drop vapor diffusion method. The optimised crystals of Mn-Sh-PPase were grown at 12 °C by mixing 0.1 μL of protein solution and 0.1 μL reservoir solution containing 30% polyethylene glycol (PEG)3350, 0.5 M NaCl, 0.1 M glycine and 0.1 M citrate (pH 6.0). Crystals of Mg-Sh-PPase in complex with PNP was obtained at 12 °C with the reservoir solution of 24% PEG3350, HEPES (pH 7.4), 10 mM ammonium phosphate dibasic and 0.2 M NaCl.

### X-ray data collection, phase determination and structure refinement

Crystals were soaked in the cryoprotectant containing reservoir solution with additional 30% glycerol. X-ray diffraction data were collected at SPring-8 beamline BL41XU using a wavelength of 1.0 Å. The diffraction images were indexed, integrated and scaled using X-ray detector software (XDS)^41^. The data collection and refinement statistics are shown in Table 2. The initial phase of the Mn-Sh-PPase crystal was obtained by the molecular replacement (MR) method. Crystal structure of family II PPase from *Methanococcus jannaschii* (PDB code 2EB0) was used as a search model in the Phaser program^42^. The initial phases of PNP and Mg-Sh-PPase were obtained by MR using the model of Mn-Sh-PPase and then automatically rebuilt using the ARP/wARP program^43^. The models were improved by multiple rounds of manual rebuilding using Coot^44^ and a restrained refinement using Refmac5^45^ in CCP4^46^ package.

**Table 2:** X-ray data collection and refinement statistics of Sh-PPase.

	Mn^2+^-bound	PNP, Mg^2+^-bound
**Data collection**		
Wavelength (Å)	1.0	1.0
Resolution (Å)	50–2.2	50–1.3
(highest shell)	2.33–2.20	1.34–1.30
Space group	*P*1	*P*2_1_
Cell dimensions		
*a/b/c* (Å)	52.53/75.57/85.67	53.49/78.98/75.3
α/β/γ (°)	107.40/90.06/92.17	90/98.59/90
Total reflections	216312	1344948
Unique reflections	61908	289618
*R*_meas_ (%)^a,b^	14.6 (74.7)	5.1 (36.6)
Completeness (%)^a^	96.8 (95.4)	96.6 (72.3)
Redundancy^a^	3.5 (3.6)	4.6 (2.6)
*I*/σ(*I*)^a^	6.9 (2.0)	18.2 (3.0)
CC_1/2_^a^	99.2 (76.4)	99.9 (90.6)
**Refinement**		
Resolution (Å)	50–2.2	50–1.3
Number of monomers/asymmetric units	4	2
*R*_work_^c^	0.256	0.096
*R*_free_^c^	0.299	0.122
RMSD^d^ bond (Å)	0.009	0.013
RMSD^d^ angle (°)	1.40	1.80
**PDB code**	6LL7	6LL8

^a^Values in parentheses are for the highest-resolution shell.

^b^*R*_meas_ = Σ_*hkl*_{*n*/(*n*−1)}^/2^ Σ_*i*_ | *I*_*i*_(*hkl*)-<*I*(*hkl*)>|/Σ_*hkl*_Σ_*i*_*I*_*i*_(*hkl*), where *n* is the multiplicity of reflection *hkl*, and <*I*(*hkl*)> is the average intensity of *i* observations.

^c^*R*_work_ = Σ_*hkl*_ | *F*_obs_(*hkl*) - *F*_calc_(*hkl*)|/Σ_*hkl*_*F*_obs_(*hkl*), where *F*_obs_ and *F*_calc_ are the observed and calculated structure factors, respectively. *R*_free_ was calculated with 5% of the reflections.

^d^RMSD, root mean square deviation.

### EPR spectroscopy

X-band EPR spectra were obtained using a Bruker ELEXSYS E580 spectrometer at the Analytical Research Centre for Experimental Sciences, Saga University. The spectra were acquired in the CW mode at ~9.597 GHz using an Oxford Instruments ESR 910 continuous helium flow cryostat. Typical experimental parameters were 2 mW microwave power, 100 kHz field modulation, 10 G modulation amplitude, ~24 G/s sweep rate and 164 ms time constant.

### EPR analysis

The di-Mn^2+^ centre has ‘ladder’ spin states from *S* = 0, 1, 2 … 5 with a weak antiferromagnetic exchange coupling (Fig. 1)^26,27^. This system can be described with spin Hamiltonian (Eq. 1) as follows:

1 $$ {\mathcal H} =-2J{S}_{1}\cdot {S}_{2}+{S}_{1}\cdot D\cdot {S}_{2}+{\sum }_{i=1}^{2}(\beta B\cdot {g}_{i}\cdot {S}_{i}+{S}_{i}\cdot {D}_{i}\cdot {S}_{i}+{S}_{i}\cdot {A}_{i}\cdot {I}_{i})$$

where *J* is the isotropic exchange coupling constant between di-Mn ions; *β* is the electron Bohr magneton; *g*_*i*_, *S*_*i*_, *D*_*i*_, *A*_*i*_ and *I*_*i*_ are the *g* tensor, electron spin operator, axial ZFS parameter, hyperfine coupling constant and nuclear spin operator for each Mn^2+^ ion, respectively. The first term is Heisenberg exchange coupling, the second term is dipolar exchange coupling, and the third term corresponds to Zeeman splitting, single ZFS, and hyperfine coupling for each Mn^2+^ ion. The spin Hamiltonian diagonalization yields all energy levels. However, calculation of the energy levels of the di-Mn^2+^ system (*S*_1_ = *S*_2_ = 5/2, *I*_1_ = *I*_2_ = 5/2) is complicated, because it requires the matrix diagonalization of 6 × 6 × 6 × 6 versus 6 × 6 × 6 × 6. Dismukes *et al*. reported an empirically linear correlation between the Mn-Mn distance from X-ray crystallography and EPR-derived ZFS parameter from the second excited state (*D*_2_) (Fig. 1)^28^. So, in this paper, the spin Hamiltonian was simplified as a single total spin system, such as *S* = 1, 2 … with two nuclear hyperfine coupling of two *I* = 5/2 (Eq. 2).

2 $$ {\mathcal H} =\beta B\cdot g\cdot S+D({S}_{z}^{2}-\frac{S(S+1)}{3})+E({S}_{x}^{2}-{S}_{y}^{2})+S\cdot {A}_{1}\cdot {I}_{1}+S\cdot {A}_{2}\cdot {I}_{2}$$

In addition, the sign of the axial (*D*_2_) and rhombic (*E*_2_) ZFS parameters for *S* = 2 were assumed to be negative and zero, respectively. The axial ZFS parameters for *S* = 1 and 2 were obtained by the best fit of the peak position with EPR simulation generated by the EasySpin 5.2.20 program^47^ operating in Matlab.

The temperature dependence of EPR signal intensity was proportional to the weight of the Boltzmann population for total spin states. The Boltzmann curves for each total spin system were calculated by Eq. 3 as follows:

3 $${\rm{Boltzmann}}\,{\rm{population}}=\frac{(2S+1)\exp \{\frac{[-2JS(S+1)-{S}_{1}({S}_{1}+1)-{S}_{2}({S}_{2}+1)]}{kT}\}}{{\sum }_{S=0}^{5}(2S+1)\exp \{\frac{[-2JS(S+1)-{S}_{1}({S}_{1}+1)-{S}_{2}({S}_{2}+1)]}{kT}\}}$$

where *J* is the isotropic exchange coupling, *S* is total spin and *S*_i_ is each Mn spin. To obtain the *J* value, the intensities of the EPR signal with temperature were plotted and normalised appropriately, and the Boltzmann curve was calculated to fit the experimental plots.

## Supplementary Information

Supplementary Information.

## References

[1] Baykov AA, Cooperman BS, Goldman A, Lahti R (1999). Cytoplasmic inorganic pyrophosphatase. Prog Mol Subcell Biol.

[2] Shintani T (1998). Cloning and expression of a unique inorganic pyrophosphatase from Bacillus subtilis: evidence for a new family of enzymes. FEBS letters.

[3] Young TW (1998). Bacillus subtilis ORF yybQ encodes a manganese-dependent inorganic pyrophosphatase with distinctive properties: the first of a new class of soluble pyrophosphatase?. Microbiology.

[4] Baykov AA, Anashkin VA, Salminen A, Lahti R (2017). Inorganic pyrophosphatases of Family II-two decades after their discovery. FEBS letters.

[5] Parfenyev AN (2001). Quaternary structure and metal ion requirement of family II pyrophosphatases from Bacillus subtilis, Streptococcus gordonii, and Streptococcus mutans. The Journal of biological chemistry.

[6] Kuhn NJ, Wadeson A, Ward S, Young TW (2000). Methanococcus jannaschii ORF mj0608 codes for a class C inorganic pyrophosphatase protected by Co(2+) or Mn(2+) ions against fluoride inhibition. Archives of biochemistry and biophysics.

[7] Ahn S (2001). The “open” and “closed” structures of the type-C inorganic pyrophosphatases from Bacillus subtilis and Streptococcus gordonii. Journal of molecular biology.

[8] Fabrichniy IP (2004). Structural studies of metal ions in family II pyrophosphatases: the requirement for a Janus ion. Biochemistry.

[9] Fabrichniy IP (2007). A trimetal site and substrate distortion in a family II inorganic pyrophosphatase. The Journal of biological chemistry.

[10] Zyryanov AB (2004). Rates of elementary catalytic steps for different metal forms of the family II pyrophosphatase from Streptococcus gordonii. Biochemistry.

[11] Kajander T, Kellosalo J, Goldman A (2013). Inorganic pyrophosphatases: one substrate, three mechanisms. FEBS letters.

[12] Ginting EL, Iwasaki S, Maeganeku C, Motoshima H, Watanabe K (2014). Expression, purification, and characterization of cold-adapted inorganic pyrophosphatase from psychrophilic Shewanella sp. AS-11. Prep Biochem Biotechnol.

[13] Ginting EL, H. CM, Motoshima K (2014). Watanabe. Functional Characteristics of Inorganic Pyrophosphatase from Psychrotroph Shewanella sp. AS-11 upon Activation by Various Divalent Cations. Asian Journal of Chemistry.

[14] Reczkowski RS, Ash DE (1992). EPR evidence for binuclear manganese(II) centers in rat liver arginase. Journal of the American Chemical Society.

[15] Kanyo ZF, Scolnick LR, Ash DE, Christianson DW (1996). Structure of a unique binuclear manganese cluster in arginase. Nature.

[16] Vainshtein BK, Melik-Adamyan WR, Barynin VV, Vagin AA, Grebenko AI (1981). Three-dimensional structure of the enzyme catalase. Nature.

[17] Khangulov SV, Barynin VV, Voevodskaya NV, Grebenko AI (1990). ESR spectroscopy of the binuclear cluster of manganese ions in the active center of Mn-catalase from Thermus thermophilus. Biochimica et Biophysica Acta (BBA) - Bioenergetics.

[18] Besio R (2013). A Mn(II)-Mn(II) center in human prolidase. Biochimica et biophysica acta.

[19] Cammack R, Chapman A, Lu W-P, Karagouni A, Kelly DP (1989). Evidence that protein B of the thiosulphate-oxidizing system of Thiobacillus versutus contains a binuclear manganese cluster. FEBS letters.

[20] Markham GD (1981). Spatial proximity of two divalent metal ions at the active site of S-adenosylmethionine synthetase. The Journal of biological chemistry.

[21] Antanaitis BC (1987). Electron paramagnetic resonance and magnetic susceptibility studies of dimanganese concanavalin A. Evidence for antiferromagnetic exchange coupling. Biochemistry.

[22] Chien JC, Westhead EW (1971). Electron paramagnetic resonance study of the interaction of yeast enolase with activating metal ions. Biochemistry.

[23] Högbom M, Andersson ME, Nordlund P (2001). Crystal structures of oxidized dinuclear manganese centres in Mn-substituted class I ribonucleotide reductase from Escherichia coli: carboxylate shifts with implications for O2 activation and radical generation. JBIC Journal of Biological Inorganic Chemistry.

[24] Pierce BS, Elgren TE, Hendrich MP (2003). Mechanistic implications for the formation of the diiron cluster in ribonucleotide reductase provided by quantitative EPR spectroscopy. Journal of the American Chemical Society.

[25] Cirino NM (1995). Divalent cation modulation of the ribonuclease functions of human immunodeficiency virus reverse transcriptase. Biochemistry.

[26] Howard T, Telser J, DeRose VJ (2000). An electron paramagnetic resonance study of Mn2(H2O)(OAc)4(tmeda)2 (tmeda = N,N,N′,N′-tetramethylethylenediamine): a model for dinuclear manganese enzyme active sites. Inorganic chemistry.

[27] Golombek AP, Hendrich MP (2003). Quantitative analysis of dinuclear manganese(II) EPR spectra. J Magn Reson.

[28] Khangulov SV, Pessiki PJ, Barynin VV, Ash DE, Dismukes GC (1995). Determination of the metal ion separation and energies of the three lowest electronic states of dimanganese (II,II) complexes and enzymes: catalase and liver arginase. Biochemistry.

[29] Geschwind, S. *Electron paramagnetic resonance*. (Plenum Publishing Corporation, 1972).

[30] Harris EA (1972). EPR of Mn2+pairs in MgO and CaO. Journal of Physics C: Solid State Physics.

[31] Coles BA, Orton JW, Owen J (1960). Antiferromagnetic Exchange Interactions between ${\mathrm{Mn}}^{2+}$ Ions in MgO. Physical review letters.

[32] Harris EA, Owen J (1963). Biquadratic Exchange Between ${\mathrm{Mn}}^{2+}$ Ions in MgO. Physical review letters.

[33] Heming M, Lehmann G, Mosebach H, Siegel E (1982). EPR investigation on exchange coupled Mn2+ pairs in (CH3)4NCdCl3. Solid State Communications.

[34] Flassbeck C (1992). Coordination of 4, 7-bis (2-hydroxybenzyl)-1-oxa-4, 7-diazacyclononane (LH2) with manganese (II) and-(III) and zinc (II). Crystal structure of [(LH) 2Zn2 (. mu.-OH)](PF6). cntdot. 0.5 CH3OH. Inorganic chemistry.

[35] Pessiki P, Khangulov S, Ho D, Dismukes GC (1994). Structural and functional models of the dimanganese catalase enzymes. 2. Structure, electrochemical, redox, and EPR properties. Journal of the American Chemical Society.

[36] Pessiki P, Dismukes GC (1994). Structural and functional models of the dimanganese catalase enzymes. 3. Kinetics and mechanism of hydrogen peroxide dismutation. Journal of the American Chemical Society.

[37] Symons, M. *Chemical and Biochemical Aspects of Electron-spin Resonance Spectroscopy*. (Van Nostrand Reinhold, 1978).

[38] Coffino AR, Peisach J (1996). Simulation of Mn(II) EPR Spectra Using a Full Spin-Hamiltonian Approach. Journal of Magnetic Resonance, Series B.

[39] Stich TA (2007). Multifrequency pulsed EPR studies of biologically relevant manganese(II) complexes. Applied magnetic resonance.

[40] Zyryanov AB, Lahti R, Baykov AA (2005). Inhibition of family II pyrophosphatases by analogs of pyrophosphate and phosphate. Biochemistry (Mosc).

[41] Kabsch W (2010). XDS. Acta Crystallographica Section D.

[42] McCoy AJ (2007). Phaser crystallographic software. Journal of Applied Crystallography.

[43] Langer G, Cohen SX, Lamzin VS, Perrakis A (2008). Automated macromolecular model building for X-ray crystallography using ARP/wARP version 7. Nature Protocols.

[44] Emsley P, Lohkamp B, Scott WG, Cowtan K (2010). Features and development of Coot. Acta Crystallographica Section D.

[45] Murshudov GN, Vagin AA, Dodson EJ (1997). Refinement of Macromolecular Structures by the Maximum-Likelihood Method. Acta Crystallographica Section D.

[46] Collaborative (1994). The CCP4 suite: programs for protein crystallography. Acta Crystallographica Section D.

[47] Stoll S, Schweiger A (2006). EasySpin, a comprehensive software package for spectral simulation and analysis in EPR. J Magn Reson.

