# Supplementary material for: X-ray Crystallography and Electron Paramagnetic Resonance Spectroscopy Reveal Active Site Rearrangement of Cold-Adapted Inorganic Pyrophosphatase
Source: Sci Rep. 2020 Mar 9;10:4368. doi: 10.1038/s41598-020-61217-6 (PMC7062746; doi:10.1038/s41598-020-61217-6)
Supplement: Supplementary file 1 — Supplementary Information. [file 41598_2020_61217_MOESM1_ESM.pdf]

X-ray Crystallography and Electron Paramagnetic Resonance Spectroscopy Reveal Active Site Rearrangement of Cold-Adapted Inorganic Pyrophosphatase

Masaki Horitani[1]*, Kazuki Kusubayashi[1], Kyoka Oshima[1], Akane Yato[1], Hiroshi Sugimoto[2], Keiichi Watanabe[1]

[1] Department of Applied Biochemistry and Food Science, Saga University, 1 Honjo-machi, Saga, Saga, 840-8502, Japan

[2] Synchrotron Radiation Life Science Instrumentation Team, RIKEN SPring-8 Center, 1-1-1 Kouto, Sayo, Hyogo, 679-5148, Japan

* To whom correspondence: horitani@cc.saga-u.ac.jp, +81-952-28-8782

This PDF file includes:

Figs. S1 to S6

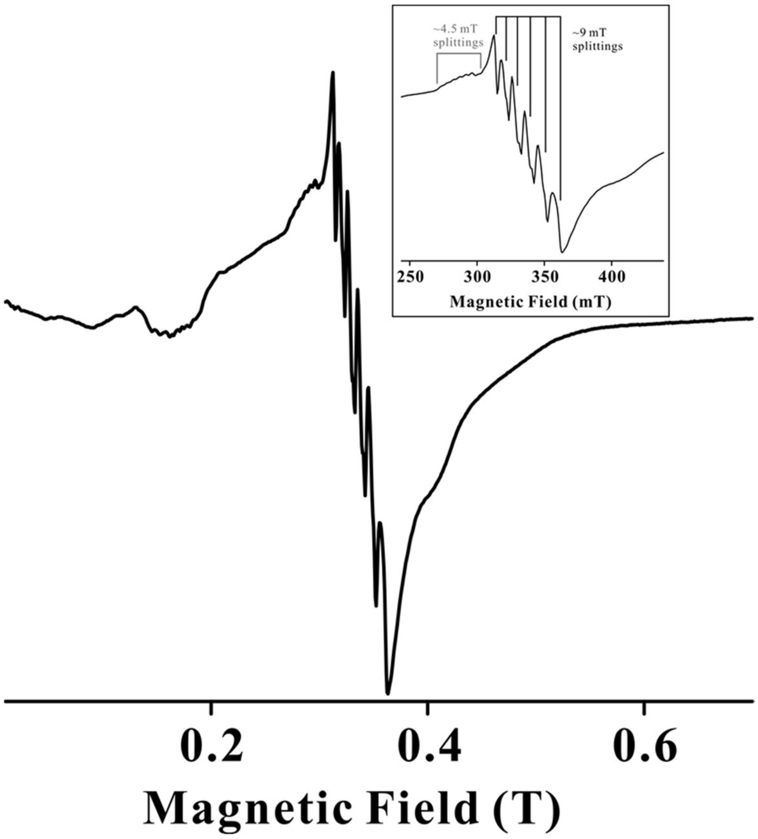

Fig S1. CW X-band EPR spectrum for Mn-Sh-PPase of initial preparation.

Mn^{2+} activated Sh-PPase was prepared by the incubation into the buffer in the presence of excess of Mn^{2+} ions at 4 °C for 2 hrs, and then removed the excess of Mn^{2+} ions by buffer exchange. Inset was expanded spectrum at around *g*~2. Six hyperfine splitting with ~9 mT were observed, which was characteristic signal of mono-Mn^{2+} ion. The signals at ~300 mT was split by ~4.5 mT, suggesting Mn-Sh-PPase also contained di-Mn center. Experimental conditions were same as Fig. 3.

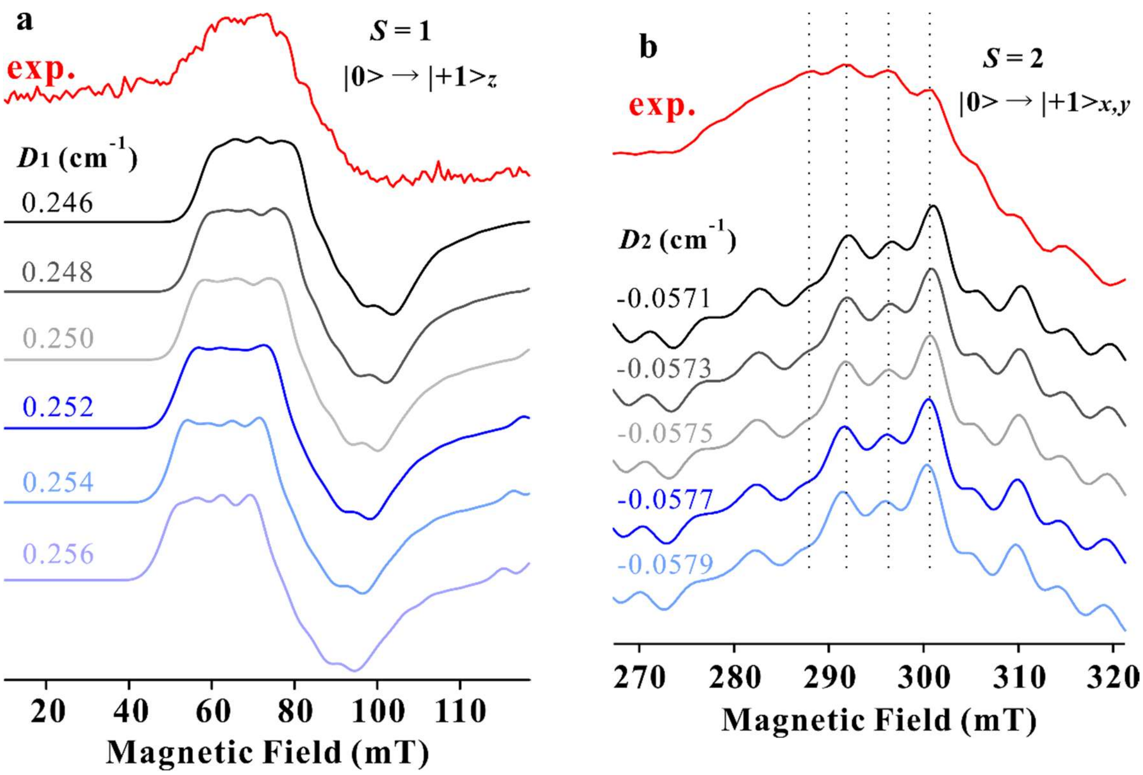

Fig S2. EPR signals and simulation from S = 1 and S = 2.

(A) Experimental EPR signal from $S = 1$ was shown in red (top). Simulation with various axial zero-field splitting (ZFS) parameters (D_1) for $S = 1$ were shown in below. Parameters were indicated in figure. The transition was assigned by simulation ($|0\rangle \rightarrow |+1\rangle_z$). (B) Experimentally assigned peak from $S = 2$ was shown in red. Simulation with various axial ZFS parameters (D_2) for $S = 2$ were shown in below. The sign of D_2 was assumed the negative and magnitude of D_2 were indicated in figure. The peak was assigned from $|0\rangle \rightarrow |+1\rangle_{x,y}$ transition by simulation.

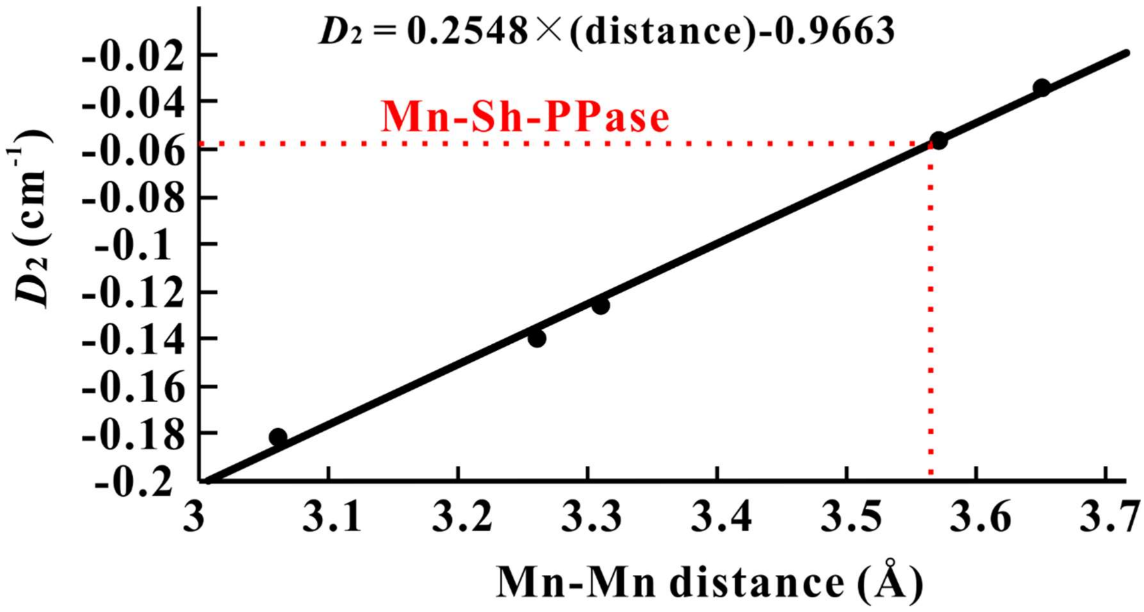

Fig S3. The empirically linear correlation between D_2 and di-Mn distance.

The data of plots was taken from points 1 to 5 of reference[1]. D_2 and distance for Mn-Sh-PPase was indicated by dot lines. The distance was calculated by equation of $D_2 = 0.2548 \times (\mathbf{di-Mn\ distance}) - 0.9663$.

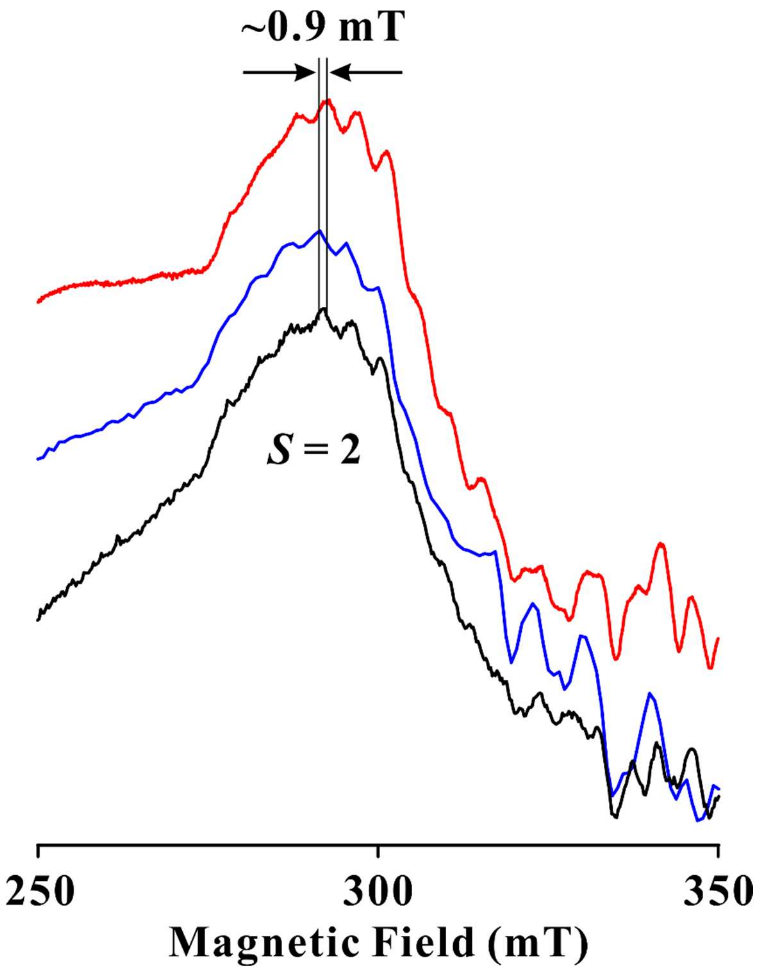

Fig S4. CW X-band EPR spectra for three Mn-Sh-PPases at 15 K.

Three samples were prepared independently. The experimental deviation of the peak position for *S* = 2 was estimated to ~0.9 mT. Experimental conditions were same as Figure 3.

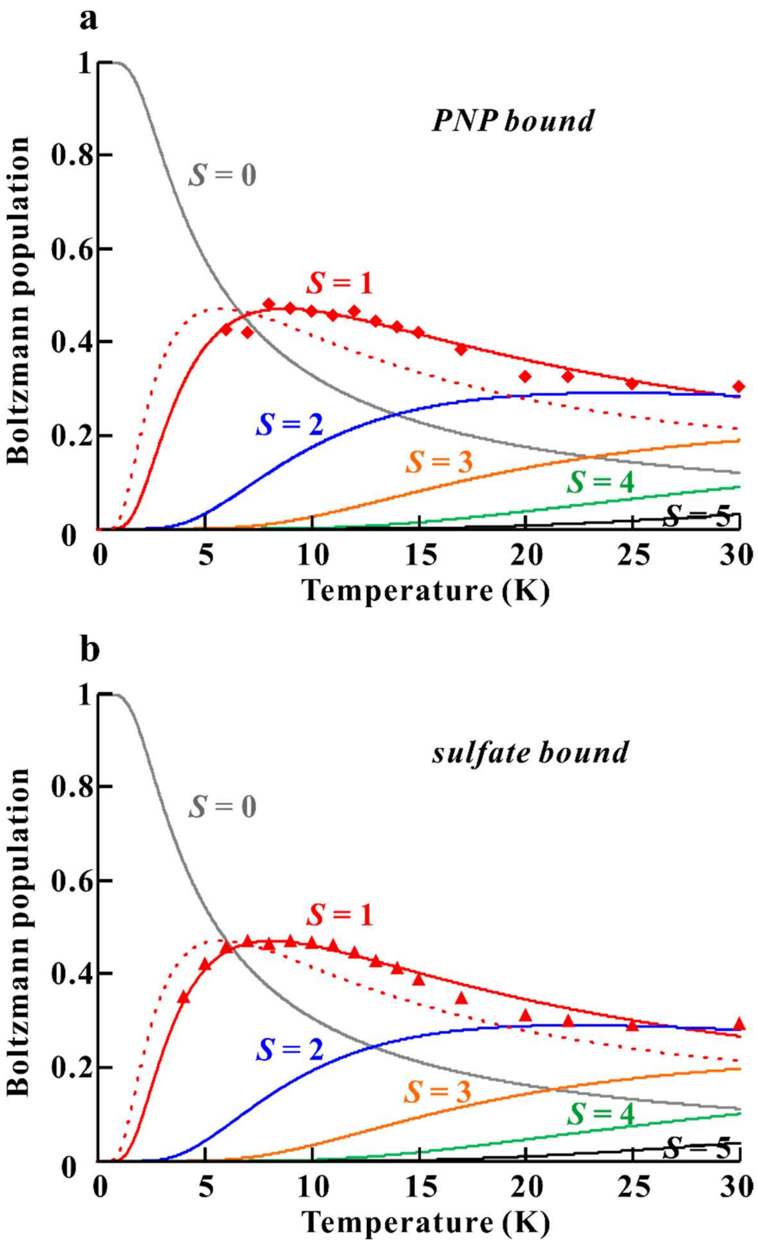

Fig S5. Overall Boltzmann curves for Mn-Sh-PPase complexed with PNP (A) and sulfate ion (B). The Boltzmann curves were shown in gray, red, blue, orange, green and black lines for S = 0, 1, 2, 3, 4 and 5, respectively. The temperature dependence of EPR signals from *S* = 1 were plotted in red diamonds and triangles for PNP bound and sulfate ion bound, respectively. The best fit exchange coupling constants for PNP bound and sulfate ion bound were J = -1.3 and -1.2 cm^{-1}, respectively.

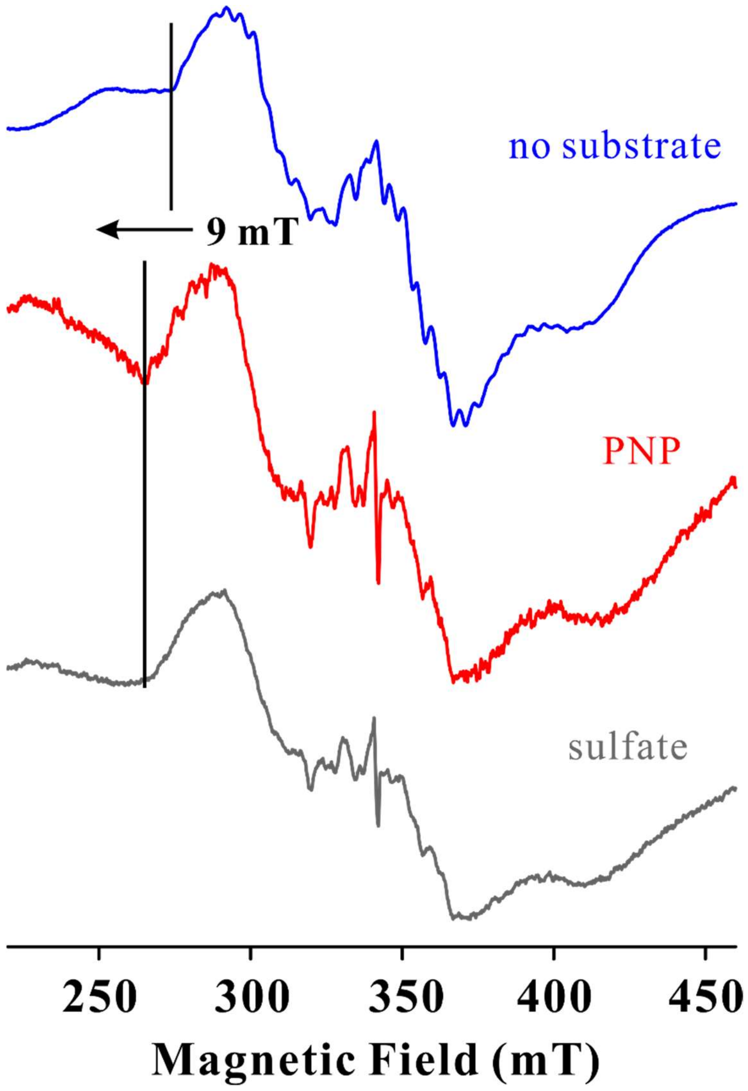

Fig S6. Comparison of EPR spectra of Mn-Sh-PPase with and without substrate analogue.
The EPR spectra of Mn-Sh-PPase without and with PNP and sulfate ion were shown in blue, red and gray lines, respectively. The expected signals of $S = 2$ transition were indicated in black lines. This peak was shift to ~9 mT lower magnetic field when both PNP and sulfate were bound with Sh-PPase. The experimental conditions were the same as Fig 3 except for temperature. The data were taken at 15 K and 40 K for no substrate and with substrate analogue, respectively.

References

1 Khangulov, S. V., Pessiki, P. J., Barynin, V. V., Ash, D. E. & Dismukes, G. C. Determination of the metal ion separation and energies of the three lowest electronic states of dimanganese (II,II) complexes and enzymes: catalase and liver arginase. *Biochemistry* **34**, 2015-2025 (1995).
